# Upcycling of Enzymatically Recovered Amino Acids from Textile Waste Blends: Approaches for Production of Valuable Second-Generation Bioproducts

**DOI:** 10.1021/acssusresmgt.4c00404

**Published:** 2025-01-03

**Authors:** Sophia Mihalyi, Irene Milani, Diego Romano, Silvia Donzella, Marion Sumetzberger-Hasinger, Felice Quartinello, Georg M. Guebitz

**Affiliations:** † Department of Agrobiotechnology, IFA-Tulln, Institute of Environmental Biotechnology, BOKU University, Vienna, Konrad-Lorenz-Strasse 20, 3430 Tulln an der Donau, Austria; ‡ Department of Food, Environmental, Nutritional Sciences (DeFENS), Università degli Studi di Milano, via Celoria 2, 20133 Milan, Italy; § acib GmbH, Konrad-Lorenz-Strasse 20, 3430 Tulln an de rDonau, Austria

**Keywords:** enzymatic recycling, protease, textile waste blends, wool/polyester, *Chlorella vulgaris*, *Rhodotorula mucilaginosa*, valuable bioproducts

## Abstract

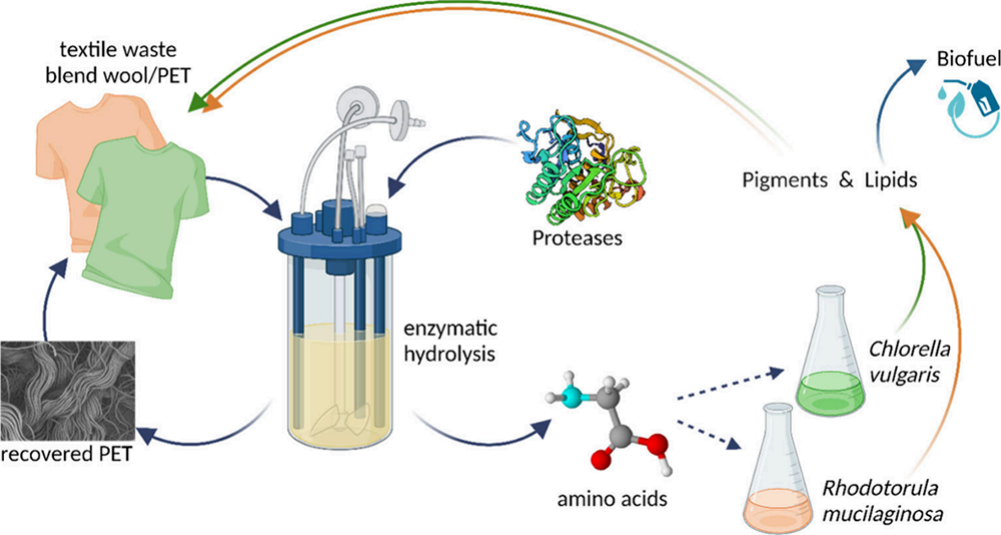

Tremendous quantities of textile waste generated and primarily landfilled annually represent a huge risk of contaminating the environment, together with loss of valuable resources. Especially, blended fabrics further pose a challenge for recycling and valorization strategies, while enzymatic hydrolysis offers a highly specific and environmentally friendly solution. In this study, we demonstrate that proteases specifically hydrolyze the wool components in blends with polyester, allowing recovery of pure polyester fibers as well as amino acids and peptides as platform molecules for further valorization. Recovered amino acids and peptides were successfully used as a nitrogen source for cultivation of *Chlorella vulgaris* and *Rhodotorula mucilaginosa* for the production of valuable biomolecules including pigments and lipids. Here, 11.3 mg/g_CDW_ chlorophyll and 47% lipid content were obtained from algal biomass, while 1.1 mg/g_CDW_ carotenoids and 35% lipids content were reached from the yeast grown on wool hydrolysate as the sole nitrogen source. These could be applied as natural dyes for textile applications or as biofuels to replace toxic synthetic compounds and fossil resources, respectively. The presented concept demonstrates feasibility of enzymatic recovery and microbial valorization of components of blended textile waste to support the development toward a circular bioeconomy.

## Abstract 2

Novel sustainable and environmentally friendly textile recycling and upcycling approaches are urgently needed to avoid increasing accumulation of nonbiodegradable polymers and toxic compounds in landfills.

## Introduction

Textile waste management is an almost unsolved sector among municipal solid waste, while textile production is expected to reach nearly 150 million tons by 2030.^1^ Textile recycling is affected by a large share of fiber blends usually comprising natural or biobased (cellulose or protein-based) and synthetic polymer-based fibers (polyethylene terephthalate (PET) or nylon). This provides beneficial properties but represents a challenge in developing recycling strategies as these interconnected fibers need to be somehow separated.^2^ Wool fibers show unique comfortable and temperature insolating characteristics, with an estimated production of 1 million tons per year. Unfortunately, wool is prone to felting during machine washing, which considerably reduces the possibility of wool mechanical recycling. Wool is often blended with polyester fibers to improve certain properties such as water repellence and shrinking.^3^ From a chemical perspective, wool is composed of 95–98% proteins where from 80–85% is keratin that has many disulfide bonds (7–20% cysteine residues) and therefore shows high stability.^4^ Besides α-keratin as a basic building block, matrix proteins of wool fibers contain high numbers of cysteine, glycine, and tyrosine residues.^5^

Complete or partial decomposition of wool has been of industrial interest for a long time to improve the characteristics of wool textiles as well as for waste treatment. This can be performed by application of chemicals, oxidizing and reducing agents, ionic liquids, or physicochemical treatments which also represents several disadvantages such as toxicity, high price, or special equipment requirement.^6−8^ Furthermore, enzymes have been used to specifically modify wool surfaces for antishrinking properties.^9^ Here, enzymatic hydrolysis was used as an environmentally friendly approach to specifically decompose one type of fiber from wool/PET blends. It has previously been demonstrated that PET recovered by enzymatic hydrolysis of blends with cotton can be regranulated and respun to fibers for textile manufacture while there are many applications for the resulting glucose.^10,11^ Likewise, PET could be directly reused after enzymatic separation of blends with wool, but much less research has been conducted on recycling and valorization of these blends so far.^2,12^ Concomitantly, enzymatic hydrolysis of the wool components yields valuable low molecular weight peptides (keratin) and amino acids.^13^ Common applications of keratin-rich waste streams are in animal feed and as fertilizers. Further concepts include cosmetic and pharmaceutical applications.^14,15^ More recently, application of keratin in (food) packaging was reported as a biobased and biodegradable alternative to conventional materials.^4^ Attempts have as well been made to regenerate fibers from hydrolyzed wool textiles using ionic liquid and blending with high molar mass cellulose.^16^ Additional approaches include the application of protein hydrolysates as flame retarder and binder.^17^ However, the hydrolysate from textile feedstock usually contains dyes and other additives that are released again during the recycling process.^18^ This could limit the application of this waste material in, e.g., feedstock and fertilizers. Therefore, two organisms that exhibit higher tolerance to toxic additives were chosen for valorization of the wool hydrolysate as a growth substrate. The microalgae *Chlorella vulgaris* and the yeast *Rhodotorula mucilaginosa* were investigated related to the production of added-value molecules. Recycling of waste wool textiles includes applications in architecture or sewage treatment as well as fertilizers, finishing agents, and regenerated protein materials.^19^ Application of enzymatic wool hydrolysate after separation from fiber blends as a nitrogen source in microbial fermentation adds a novel approach in urgently needed waste wool textile recycling.

Microalgal biomass represents a valuable source for a variety of biomolecules such as pigments, proteins, lipids, polysaccharides, and vitamins. Among microalgae, *C. vulgaris* has been investigated for growth in the presence of inhibiting compounds and for biodegradation of dyes in wastewater treatment showing promising results.^20−24^ Besides heterotrophic growth, microalgae like *C. vulgaris* can also grow on CO_2_ as a carbon source performing photosynthesis and using natural light as an energy source and are therefore assessed for sustainable bioproduction. Nevertheless, a nitrogen source is required that can be inorganic (NO_3_, NO_2_, NO, NH_4_) or organic (urea, amino acids) and can represent high cost.^25^ Therefore, nitrogen-rich wool hydrolysate could serve as both an economically attractive and sustainable nitrogen source for growth of *C. vulgaris*.

The yeast *R. mucilaginosa* was previously studied for wastewater treatment containing dyes and therefore represents another promising organism for valorization of textile waste hydrolysate.^26,27^*R. mucilaginosa* can naturally produce carotenoids which are widely employed in various industrial sectors (i.e., food and feed industry, nutraceutical, pharma).^28^ Yeasts synthesize carotenoids and lipids with high yields when cultivated on synthetic media.^29^ Moreover, oleaginous red yeasts are capable of efficiently metabolizing a wide range of carbon sources, as reported in many studies, where they have been found suitable to produce bioproducts from different types of waste and residues.^30,31^

In this study, we have investigated the potential of a nitrogen-rich hydrolysate resulting from enzymatic separation of wool/PET blended textiles as the nitrogen source for the cultivation of two microorganisms, namely, *Chlorella vulgaris* and *Rhodotorula mucilaginosa*. These organisms can produce valuable pigments and lipids that could potentially be applied in the textile industry or as biofuel as an eco-friendly alternative to synthetic dyes and fossil resources, respectively.

## Materials and Methods

### Materials, Chemicals, Enzymes, and Organisms

The 40% wool/60% polyethylene terephthalate (WO/PET 40/60) blend was purchased from Textil Müller GmbH (Kritzendorf, Austria). Savinase 12T protease enzyme was purchased from Novozymes (Copenhagen, Denmark). *Chlorella vulgaris* 211-116 was from the culture collection of FHWN, Campus Tulln, Austria. *Rhodotorula mucilaginosa* Ex7, available in the UBO Culture Collection (https://www.univ-brest.fr/ubocc/fr), was used for yeast cultivation experiments. All other chemicals and solvents were used without further purification and purchased from Sigma-Aldrich (Vienna, Austria) or Carl Roth (Germany) unless stated otherwise.

### Wool Hydrolysis from Wool/PET Blends

The WO/PET blend was first milled to a size ≤6 mm. THen, 75 g of WO/PET was added to 1 L of 50 mM Tris/HCl buffer pH 9 containing 2% of protease stock (0.85 U/mL, 1.1 mg/mL). The hydrolysis reaction was performed at 50°C for 96 h in triplicate to characterize the hydrolysis process and record the plateau of amino acid concentration. The supernatant was sterile filtered through a 0.2 μm PES filter to avoid contamination during cultivation of microorganisms.

### Quantification of Amino Acids

#### Ninhydrin Assay

Primary amino groups were detected by the ninhydrin reaction that forms a blue dye in alkaline solution with glycine calibration from 0–200 μM. THen, 75 μL of ninhydrin reagent (7.5 mg hydrindantin and 50 mg ninhydrin in 1.875 mL DMSO and 625 μL of 4 M Na-Acetate buffer pH 5.2) was added to 100 μL of the sample, vortexed, and incubated at 80 °C for 30 min. After cooling, 100 μL of stabilizing solution (50% ethanol) was added, vortexed, and centrifuged for 5 min at 12700 rpm (Eppendorf Centrifuge 5427 R). Then, 200 μL was transferred to a 96-well plate, and absorbance was measured at 570 nm on an Infinite 200 Pro spectrophotometer (Tecan, Switzerland).

#### Phenol Content Assay

Phenol group content was determined by using the Folin-Ciocalteau (FC) assay by formation of a blue phosphotungstic-phosphomolybdenum complex that can be quantified by UV–vis spectrophotometry.^32^ Calibration was performed with vanillin from 0.05–1 g/L. Then, 60 μL of FC-reagent and 600 μL of ultrapure water were added to 20 μL of the sample, vortexed, and incubated for 5–8 min at 21 °C. Afterward, 200 μL of 20% Na_2_CO_3_ solution and 120 μL of ultrapure water were added, vortexed, and shaken for 2 h at 21 °C and 800 rpm. Then, the absorbance was measured at 760 nm in a 96-well plate.

#### Total Carbon and Nitrogen Content

For the determination of total dissolved carbon (TC), the sample was catalytically combusted and the developed CO_2_ measured with NDIR. The catalyst, platinum-coated aluminum oxide pearls, was heated to 720 °C. The total carbon comprises the organic and inorganic carbon in the sample. For the standard stock solution, 2.125 g of potassium hydrogen phthalate was dissolved in 1 L of ultrapure water (Arium, Sartorius, Göttingen, Germany) resulting in a concentration of 1000 mg C/L. Measurement of total dissolved nitrogen bonded (TN_b_) reflects the amount of total nitrogen in the sample in the form of ammonia, nitrate, and nitrite, as well as organic compounds. The sample was catalytically combusted at 720 °C, and the resulting gas was analyzed with a chemoluminescence detector. A solution of 7.219 g of KNO_3_ (1000 mg/L TN) in 1 L of ultrapure water (Arium, Sartorius, Göttingen, Germany) was used as a standard stock solution. The samples were measured with a TOC-V_CPH_ instrument equipped with an ASI-V autosampler (Shimadzu, Kyoto, Japan). For the detection of TN_b_, a TNM-1 (Shimadzu, Kyoto, Japan) was used. Oxygen 4.5 (Messer, Gumpoldskirchen, Austria) was used as the carrier gas. Samples were filtered with 0.45 μm Aquatron filters (Whatman Gemany, Göttingen, Germany) prior to analysis and diluted with ultrapure water to fit the calibration range. The instrument was calibrated with every sequence up to 200 mg/L. Determination and detection limits were calculated according to DIN 32645 for every calibration.

#### High Performance Liquid Chromatography (HPLC)

The single amino acids were identified and quantified through HPLC analysis on a 1260 series (Agilent technologies, USA) equipped with a 1290 series ELSD (Agilent Technologies, USA) as previously described.^33^ AAS18 Amino Acid Standard (Sigma-Aldrich, Austria) was used for quantification at a concentration from 50–1250 μM.

#### Microbial Cultivation

##### *Chlorella vulgaris*

*C. vulgaris* that has the GRAS (Generally Recognized As Safe) status was grown in 500 mL shake baffled flasks containing 100 mL of media at room temperature and 100 rpm under natural sunlight for 28 days on a GFL 3020 orbital shaker. Samples were taken at regular timepoints at day 0, 4, 7, 10, 14, 18, 21, 25, and 28. All cultivations were performed in biological duplicates. Optical density (OD) was measured at 750 nm on a DR3900 spectrophotometer (Hach Lange, Austria). To confirm the absence of potentially contaminating microorganisms in the cultivations, 20 μL of the cultures were plated on agar plates for each timepoint. Samples were analyzed for phenol and ninhydrin content after biomass removal by centrifugation and sterile filtration through a 0.2 μm filter. TC and TN contents were determined from the initial media after 14 days and the final cultivation supernatant after 28 days.

Gorham’s medium for algae (ATCC culture medium 625) was used for cultivation of *C. vulgaris* containing per liter 496 mg of NaNO_3_, 39 mg of K_2_HPO_4_, 75 mg of MgSO_4_·7H_2_O, 36 mg of CaCl_2_·2H_2_O, 6 mg of FeCl_3_·6H_2_O, 58 mg of Na_2_SiO_3_·9H_2_O, 20 mg of Na_2_CO_3_, 6 mg of citric acid, 1 mg of NaEDTA, and 100 μL trace element solution containing per liter 0.5 g of H_3_BO_3_, 0.04 g of CuSO_4_·5H_2_O, 0.1 g of KJ, 0.33 g of FeCl_3_·6H_2_O, 0.4 g of MnSO_4_·H_2_O, 0.2 g of (NH_4_)_6_Mo_7_O_24_·4H_2_O, and 0.4 g of ZnSO_4_·7H_2_O adapted to a pH of 7.5 ± 0.5. For agar plates, an additional 10 g/L of peptone, 10 g/L of glucose, 15 g/L of agar, and 10 mL/L of vitamin stock containing 330 mg/L of biotin, 5 mg/L of vitamin B12, and 5 mg/L of thiamin were added. For cultivations with WH as nitrogen source, 2x concentrated media without addition of NaNO_3_ was prepared. The required ratio of wool hydrolysate (WH) addition was calculated from TN measurements and diluted accordingly with concentrated media. Cells were visualized on an Olympus BX43 microscope.

##### *Rhodotorula mucilaginosa*

For long-term storage, Ex7 strain belonging to *Rhodotorula mucilaginosa* ssp was maintained at −80 °C on 15% (v/v) glycerol and 85% (v/v) YPD (10 g/Lof yeast extract, 20 g/L of peptone, and 20 g/L of glucose).

As control medium, a defined minimal mineral medium (YNB), containing 2% glucose (w/v, Sigma-Aldrich, Italy), 1.7 g/L of yeast nitrogen base (YNB, Difco, Italy), and 0.1 M 2-(N-Morpholino) ethanesulfonic acid (MES, Sigma-Aldrich, Italy) at pH 6 was used.

As a control medium for microbial lipid production, the lipidogenic (B) medium containing 20 g/L of glucose, 1 g/L of KH_2_PO_4_, 0.05 g/L of MgSO_4_·7H_2_O, 0.01 g/L of NaCl, 0.01 g/L of CaCl_2_, 1 g/L of yeast extract, and 1 g/L of (NH_4_)_2_SO_4_ was used.

For cultivations with WH as the nitrogen source, the hydrolysate was used at a final concentration of 0.1 g/L of TN and supplemented with 20 g/L of glucose. This medium was also supplemented with 0.1 M 2-(N-morpholino) ethanesulfonic acid to maintain the pH of 6. In a subsequent scale-up in the bioreactor, the MES addition can be avoided as the pH can be automatically controlled.

Submerged cultures were performed at 28 °C in 500 mL baffled flasks using 100 mL of medium, under shaking (150 rpm; INFORS HT, Multitron Standard). Precultures were prepared by inoculating cells from the glycerol stocks in baffled flasks with an air-to-liquid ratio of 5:1 overnight. Cells from precultures grown in YPD were harvested during the exponential growth phase by centrifugation and inoculated at OD_660_ 0.1. All cultures were performed in triplicates.

The yeast growth was monitored by collecting samples at regular time points and analyzing them for OD, cell dry weight (CDW), lipid content, and carotenoid content.

The increase in the OD at 660 nm was measured using a spectrophotometer (Eppendorf, Milan, Italy).

For CDW determination, cells were collected from 2 mL of culture by centrifugation (10 min at 13200 rpm in an Eppendorf 5415D centrifuge) and washed twice with deionized water. The pellets were dried at 105°C.

Glucose concentrations during the fermentation processes were determined spectrophotometrically by using a commercial enzymatic kit (K-GLUHK, Megazyme, Wicklow, Ireland).

#### Biomolecule Production

##### Chlorophyll from *C. vulgaris*

The chlorophyll content was determined by extraction from 2–4 mg of freeze-dried biomass in 1 mL of 90% methanol after washing with 1 mL of ultrapure water and incubated in the dark at room temperature overnight. The supernatant was collected by centrifugation (10 min, 12700 rpm, Eppendorf Centrifuge 5427 R) and absorbance measured at 663 nm on a DR3900 spectrophotometer (Hach Lange, Austria). The chlorophyll a content was calculated through eq 1.^34^
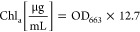
1

##### Lipids from *C. vulgaris*

To determine the lipid content, extraction of 40 mg of freeze-dried biomass was performed on an EDGE Solvent extraction device (CEM, Germany) with chloroform:methanol 2:1 for two cycles of 5 min hold time, 150 °C, and 10 mL; one cycle of 3 min, 150 °C, and 5 mL; and two washing cycles of 30 s, 150 °C, and 5 mL resulting in a total extraction volume of 35 mL. Afterward, the solvent was evaporated, samples dried at 100 °C for 1 h, and the weight of the lipid was recorded. For GC analysis, fatty acids were derivatized by sequential addition of 2 mL of 85% MeOH/15% H_2_SO_4_ and chloroform with 1 g/L of methyl benzoate as the internal standard, vortexed, and heated to 100 °C for 2 h. After cooling to room temperature, 1 mL of ultrapure water was added and vortexed, and the lower organic phase was filtered through anhydrous Na_2_SO_4_ and Na_2_CO_3_ into a glass vial. GC analysis was performed as previously described^33^ on a 7890A GC-FID (Agilent technologies, USA).

##### Carotenoids from *R. mucilaginosa*

The carotenoids concentration was determined after freezing (−20 °C) cell pellets obtained from 500 μL of culture broth and adapting the protocols from refs (35) and (36). Briefly, carotenoids were extracted by adding 500 μL of glass beads and 500 μL of hexane:ethyl acetate (50:50 (v/v)) containing 0.05% (w/v) of butyrate hydroxytoluene. This mixture was vigorously mixed in a beat beater (Precellys Evolution from VWR) at 5 °C for five cycles of 30 s at 6000 rpm with a 30 s pause. The extract was collected after 5 min centrifugation at 13000 rpm, and the extraction procedure was repeated until the pellet was colorless. The extract was then dried under nitrogen and resuspended in DMSO.

The total carotenoid concentration in the mixture was calculated by measuring absorbance at 450 nm after extraction. The concentrations were calculated through a standard curve using β-carotene from Sigma-Aldrich dissolved in DMSO as a reference. The absorbance value was correlated to the CDW measurement for each corresponding culture.

##### Lipids from *R. mucilaginosa*

The lipid content was determined via the sulfo-phosphovanilline colorimetric method (Spinreact, Girona, Spain) from washed cell pellets (≈OD 30 suspended in 0.5 mL of cold deionized water).

## Results and Discussion

### Enzymatic Hydrolysis of Wool from Blends

**Figure 1:**
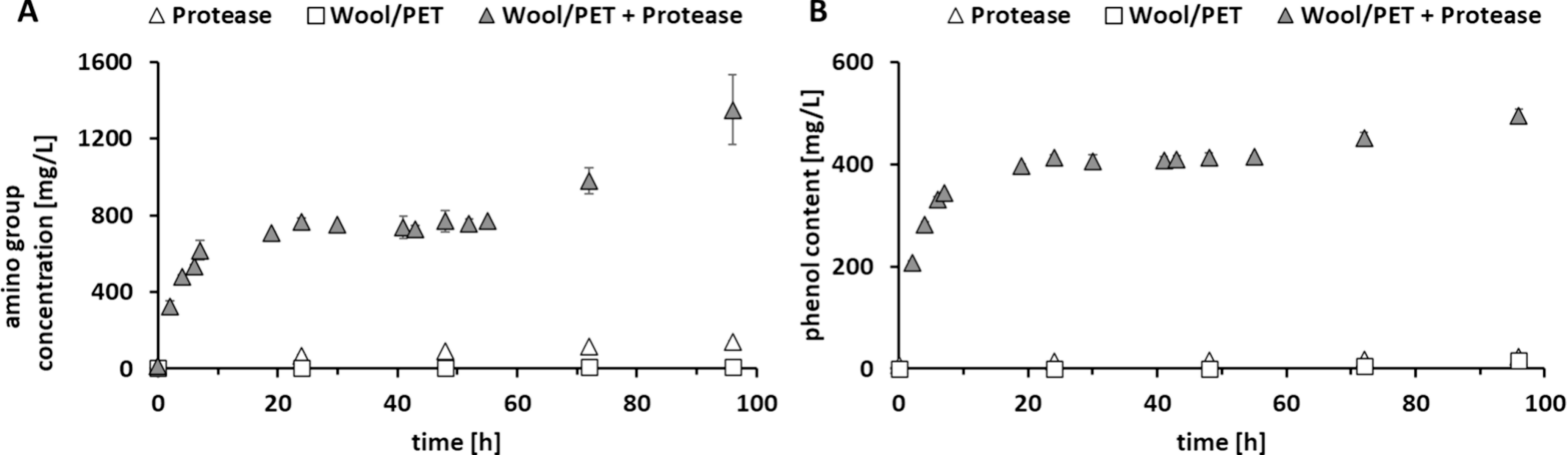
Increase in amino group content (A) and phenol content (B) during enzymatic hydrolysis of wool from WO/PET textile blends.

To enable recycling of blended fabrics, different fiber types need to be separated. Here, wool was specifically hydrolyzed by a protease in wool/PET textiles. The release of amino acids and oligopeptides during hydrolysis was monitored through the ninhydrin and phenol content assay (Figure 1). In comparison to the blanks that contained either the enzyme only (protease) or the textile (wool/PET) only, the concentration of amino groups increased significantly over time, resulting in 1350.1 ± 181.9 mg/L after 96 h together with 495.1 ± 12.7 mg/L phenol content. However, apart from phenolic amino acids (i.e., tyrosine), the phenol content could also comprise aromatic textile dyes possibly present in the hydrolysate after decomposition of dyed natural fiber.^37^ Additionally, the TC and TN contents of the hydrolysate were determined which resulted in 2466.7 ± 149.7 and 562.8 ± 18.8 mg/L, respectively, after subtraction of the blank, which indicated released concentration of carbon and nitrogen containing molecules into solution.

For (thermo-)mechanical recycling of polyester, the fibers are required to be free of contaminants. Therefore, after enzymatic hydrolysis of the wool, the purity of the recovered PET fibers was evaluated through FTIR analysis which confirmed that all wool was removed by the enzymatic process (Figure S1). The characteristic peaks at 3295, 1651, and 1519 cm^–1^ that correspond to peptide bonds from wool were not detected in the recovered PET fibers.^38^ The process could also be improved in terms of time by optimized protease formulations and applied in combination with removal of other contaminants for recovery of white polyester.^3^ Additionally, novel enzymes are still waiting to be discovered in nature, and protein engineering can be applied as a modern tool to specifically improve the performance and stability of enzymes.^39^ Furthermore, future work will include the application of statistical tools to optimize the process parameters.^40^

The extended process time was investigated to record the long-term behavior. It is apparent that wool hydrolysis is already completed after around 24 h; however, the amino group concentration continued to increase after 72 h, which indicated the potential cleaving of larger oligopeptides present in solution into smaller peptides and/or finally amino acids. This hypothesis was supported by analysis of the amino acids present in the hydrolysate over time (Figures S2–S5). Predominantly identified amino acids include threonine, valin, methionine, phenylalanine, and tyrosine together with glycine, leucin, ISO-leucin, histidine, and arginine (Figure S6) resulting in a total concentration of 1080 and 374 mg/L aromatic amino acids after 96 h (Table S1). Concentrations of amino acids still increased after 48 h reaction time together with decreasing peak areas that can represent dimers, trimers, or oligomers especially around the retention times of phenylalanine and tyrosine (Figure S5) which indicated that hydrolysis is still ongoing in solution after depletion of solid substrate.

### Upcycling of Wool Hydrolysate

Different valorization routes for WH from the textile recycling process were investigated to find new applications for amino acids and peptides recovered from textile waste. The first approach represents growth of *Chlorella vulgaris* for production of valuable compounds including chlorophyll and lipids. *C. vulgaris* can use CO_2_ as the carbon source and potentially the WH as the nitrogen source. Furthermore, *Rhodotorula mucilaginosa* was cultivated directly in WH supplemented with glucose for the production of carotenoids and lipids.

### Cultivation of *Chlorella vulgaris*

**Figure 2:**
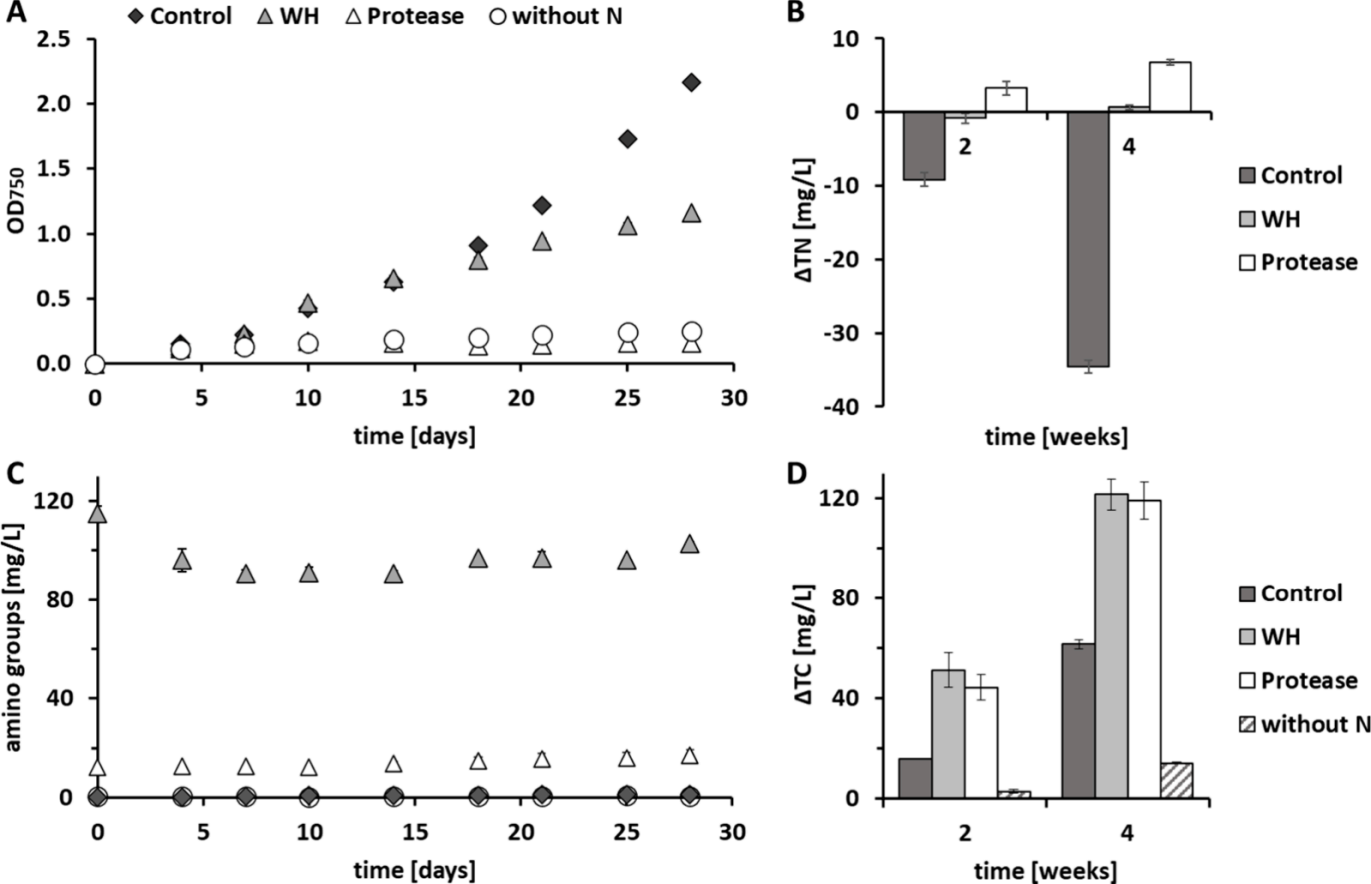
(A) OD measurements during cultivation of *C. vulgaris* on media supplemented with wool hydrolysate (WH) as nitrogen source in comparison to standard cultivation medium (control) and blanks. (B) Total nitrogen (TN) consumption after 2 and 4 weeks of cultivation of *C. vulgaris*. (C) Amino group concentration. (D) Total carbon (TC) content from the cultivation supernatant after 2 and 4 weeks.

Application of WH as a nitrogen source for growth of microalgae under natural light with CO_2_ as a carbon source showed significant biomass formation resulting in 0.48 ± 0.02 g/L under standard conditions and 0.29 ± 0.02 g/L with WH as sole nitrogen source after 4 weeks. To confirm that *C. vulgaris* does not grow without a nitrogen source, two blank conditions were performed (without any nitrogen and with only the protease solution in buffer supplemented). The results showed that almost no growth was detected without adding either nitrogen or the WH which indicates the essentiality of this component as well as confirms the possibility of utilization of WH for microalgal growth (Figure 2A). However, growth was limited over time in comparison to the standard medium (control), reaching 54% of the OD which might be caused by the nature of the nitrogen source or also the presence of inhibiting compounds such as the dyes released during the fiber hydrolysis process. Nevertheless, *C. vulgaris* has been reported previously to be applicable for textile wastewater treatment which would have an additionally beneficial impact. By increasing the WH content 10x, 63% OD of the control was reached, which indicated the impact of the nitrogen source rather than the inhibiting effect of dyes. Limiting the nitrogen source in the standard media to 10% similarly resulted in 58% growth reduction and indicated lower nitrogen availability in the form of amino acids and peptides than sodium nitrate. Previous research did not show any negative impact of present additives including dyes on growth of various organisms.^41−43^

Analysis of the soluble amino group as well as TC and TN before, during, and at the end of the cultivation showed that the amino group as well as the total nitrogen concentration did not decrease significantly during cultivation (Figure 2B, C) on WH. This indicated that although *C. vulgaris* is utilizing the WH for growth, it might at the same time secreting nitrogen-containing metabolites that lead to a rather stable nitrogen and amino group concentration in the supernatant. This is also visible from the TC content that shows a higher increase in the presence of the WH (Figure 2D). It has been reported in previous research that external factors such as the presence of dyes, e.g., can induce secretion of various metabolites including amino acids in *C. vulgaris*.^44,45^

### Extraction of Biomolecules

**Figure 3:**
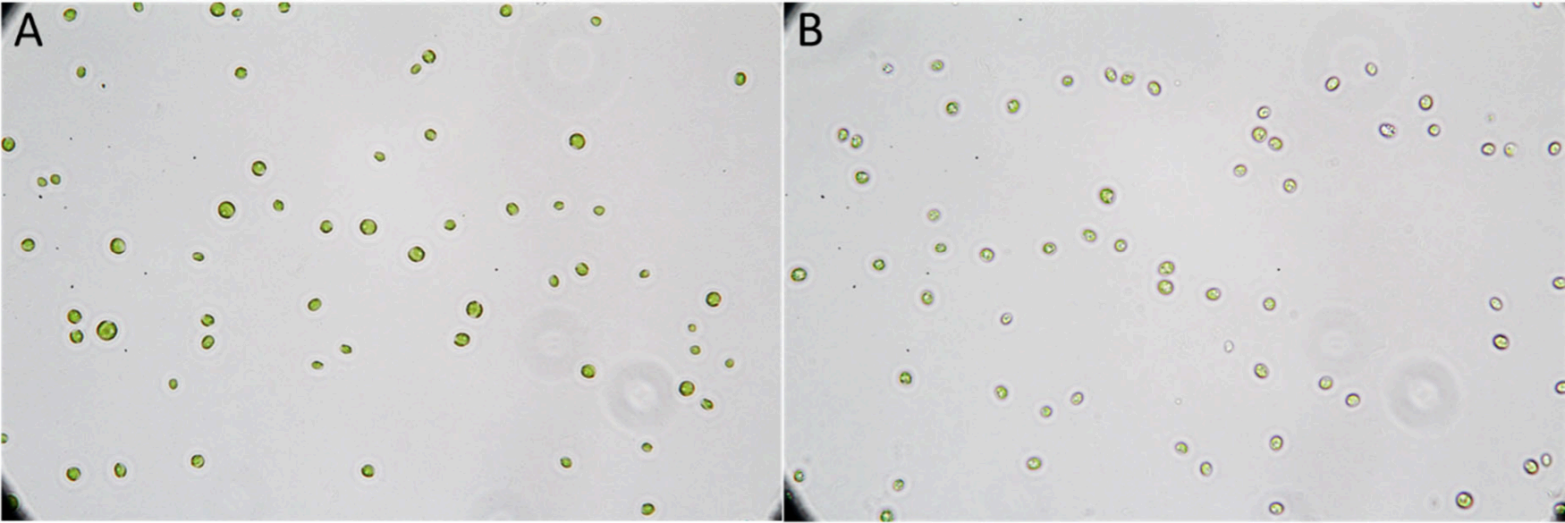
*C. vulgaris* cells captured through light microscopy with 1000× magnification grown (A) in standard medium and (B) with WH as the nitrogen source.

*C. vulgaris* can accumulate up to 7% chlorophyll of the CDW and therefore is used as a prominent industrial natural pigment producer.^46^ The extracted chlorophyll concentration resulted in 37.9 ± 8.5 mg/g_CDW_ in the control culture and 11.3 ± 0.4 mg/g_CDW_ from the culture grown on WH after 14 days and 33.7 ± 3.6 and 5.4 ± 0.2 mg/g_CDW_ after 28 days, respectively. As also the OD_750_ was lower during growth on WH, a lower chlorophyll concentration was expected. Furthermore, investigating cell morphology through light microscopy, cells grown in the presence of WH also exhibited less chlorophyll than the control (Figure 3). A possible explanation for reduced chlorophyll production could be the presence of textile dye as this can represent a stress condition for the cells or the nitrogen source that is essential for chlorophyll synthesis and growth. Chlorophyll is a nitrogen-rich compound that could also be used as an intracellular nitrogen pool^47^ which would provide an explanation for decreasing chlorophyll concentration from week 2 to week 4.

**Figure 4:**
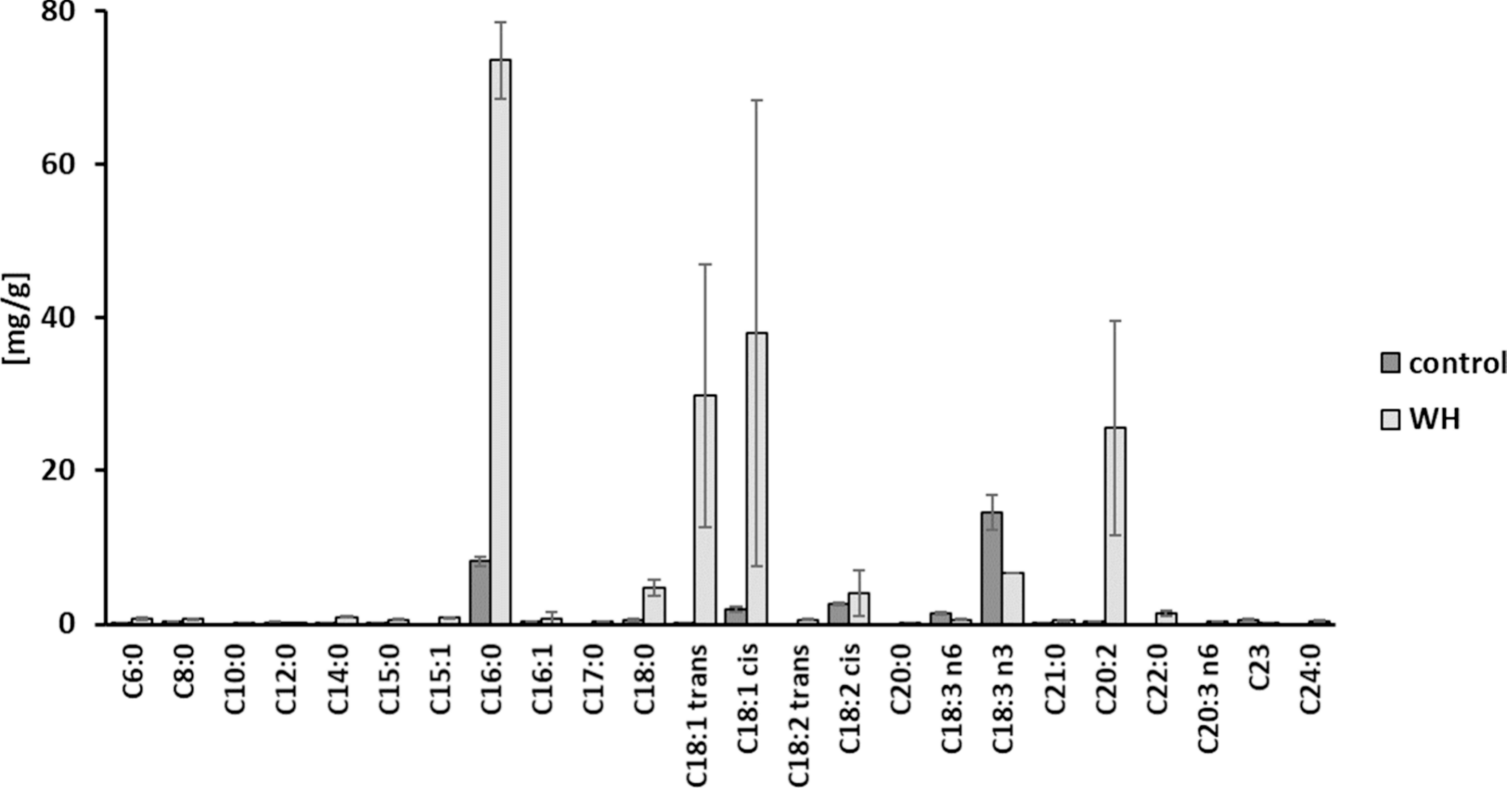
Extracted fatty acids (FAs) from *C. vulgaris* biomass in milligrams of FA per gram of initial freeze-dried biomass after cultivation in standard media (control) and WH.

On the other hand, nitrogen and salt stress conditions can lead to an increase of lipid accumulation^47^ which was indeed revealed. Here, 15.4 ± 0.9% of lipid content was obtained in the reference culture whereas 47.4 ± 0.8% was present in the biomass grown on WH which is impressive also in comparison to literature.^48,49^ To evaluate the impact of the nitrogen source on the fatty acid profile, a gas chromatography analysis was performed after lipid extraction. The fatty acid profile is further important for application of lipids as biofuel,^50^ where *Chlorella* represents a promising source of useful fatty acids such as hexadecenoic, heptadecanoic, and octadecanoic acids.^49,51^*C. vulgaris* biomass grown on WH showed 192.0 ± 38.0 mg/g total FA content, in contrast to 32.5 ± 2.4 mg/g in the control. Therefore, the presence of mainly C_16_, C_18_, and C_20_ chain length FAs were identified (Figure 4). These FA together with high lipid content are required for biofuel production proving that this biomass represents a promising source.^49^

### Cultivation of *Rhodotorula mucilaginosa*

**Figure 5:**
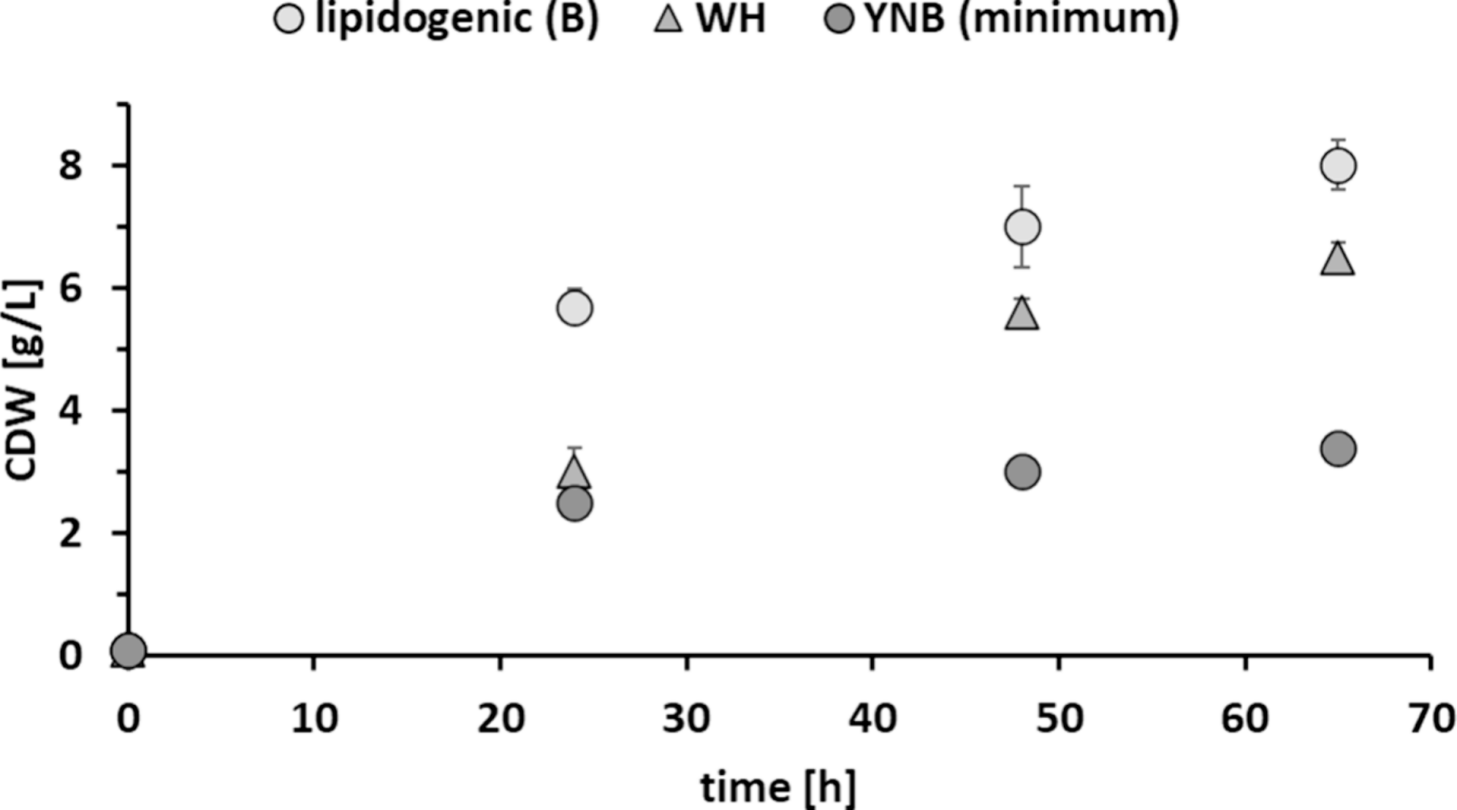
Biomass growth of *R. mucilaginosa* on lipidogenic (B), minimal YNB, and WH media.

As a second valorization opportunity for recovered amino acids, *R. mucilaginosa* was cultivated on WH as well as minimal mineral medium (YNB) and lipidogenic medium (B) as a control. In comparison to the YNB, the WH resulted in higher biomass production of 6.5 ± 0.23 g/L CDW after 65 h of cultivation (Figure 5) versus 3.4 ± 0.08 g/L in YNB. WH, being a source of already available amino acids, was sufficient to fully support the nitrogen requirement during yeast growth even more efficiently than YNB. Furthermore, no inhibition was observed due to dyes or other molecules present in the WH. In terms of biomass production, the B medium resulted in slightly higher CDW (8.0 ± 0.4 g/L) due to the presence of yeast extract, which is known to enhance yeast growth, especially in the initial growth phase (Figure 5).

Regarding lipid accumulation, after 65 h of growth, the percentage of triacyl glycerides (TAGs) in the total CDW was similar in B and WH medium, resulting in 38% (3.0 ± 0.3 g/L) and 35% (2.3 ± 0.17 g/L), respectively. Glucose quantification revealed that the available carbon source (20 g/L of glucose) was almost entirely consumed in the B medium, while a residual of 5 g/L of glucose remained in WH medium after 65 h. These data indicated that the WH can be used as a nitrogen source also in lipid production processes without significantly affecting the final yield.

Finally, *R. mucilaginosa* cells grown in WH for 65 h were analyzed for their carotenoid content, resulting in 7.4 ± 0.2 mg/L, corresponding to 1.1 mg/g_CDW_. The total carotenoid content, in line with the production reported in isolated strains of *R. mucilaginosa*,^52−54^ is very promising considering the inclusion of a waste material and its possible optimization in a bioreactor through a specific fed-batch strategy.

Carotenoids are 40-carbon-long terpenoid pigments formed by a polyene chain consisting of 9–11 double bonds and mainly terminating in rings. Their chemical structure gives them the ability to act as membrane-protective antioxidants, scavenging oxygen and peroxyl radicals.^55^ Due to the high number of conjugated double bonds, carotenoids are also natural colorants ranging from yellow to orange and red to purple.^56^ Carotenoids produced by oleaginous yeasts have the advantage of being stored inside lipid bodies increasing their bioaccessibility and avoiding the loss of their nutritive and biological desirable properties due to oxygen and light exposure.^57,58^ Regarding the expanding market for natural pigments and the wide range of applications including food, feed, and textiles, several companies are currently investing in technologies for the biotechnological production of these compounds with a significant potential to use yeasts.^59,60^

Synthetic dyes that are applied in the textile industry represent a serious concern for the environment. The textile industry is one of the most polluting sectors with around 200,000 tons of toxic dyes ending up in effluents each year.^18^ These include various kinds, such as azo, direct, reactive, acidic, and basic, which as well contain heavy metals like mercury, chromium, cadmium, and lead.^27^ Therefore, for an environmentally friendly and circular bioeconomy vision, chlorophyll and carotenoids could be used as potential substitutes for the currently applied partially toxic dyes. Additionally, natural dyes show different advantageous properties such as UV protection, antimicrobials, and antioxidant,.^25,46,61−63^

## Conclusion and Outlook

In this study, a recycling and valorization strategy for blended textile waste of wool/PET is presented. Natural fiber components of the blends were enzymatically hydrolyzed into their corresponding amino acids and oligopeptides, obtaining pure recovered synthetic fibers. The hydrolysate was applied as a valorization platform for growth of *C. vulgaris* and *R. mucilaginosa*. Valuable pigments and lipids could be extracted from the generated biomass, resulting in 11.3 mg/g_CDW_ chlorophyll and 1.1 mg/g_CDW_ carotenoids as well as 47% and 35% lipid content, respectively. Natural pigments and extracted lipids could replace toxic synthetic dyes and reduce consumption of fossil fuels in the future.

## Supporting Information Available

FTIR spectra after enzymatic hydrolysis of wool from WO/PET textile waste blends (Figure S1). HPLC chromatograms from amino acid analysis of wool hydrolysis (Figures S2–S5). Amino acid concentrations during wool hydrolysis from HPLC analysis (Figure S6, Table S1) .(PDF)

## Abbreviations

PET: polyethylene terephthalate
WO: wool
WH: wool hydrolysate
TC: total carbon
TN: total nitrogen
FA: fatty acid
YNB: minimal mineral medium
B: lipidogenic medium
CDW: cell dry weight
