# Supplementary material for: Upcycling of Enzymatically Recovered Amino Acids from Textile Waste Blends: Approaches for Production of Valuable Second-Generation Bioproducts
Source: ACS Sustain Resour Manag. 2025 Jan 3;2(1):157–65. doi: 10.1021/acssusresmgt.4c00404 (PMC11770743; doi:10.1021/acssusresmgt.4c00404)
Supplement: Supplementary file 1 — rm4c00404_si_001.pdf [file rm4c00404_si_001.pdf]

Upcycling of enzymatically recovered amino acids from textile waste blends: approaches for production of valuable second-generation bioproducts

Sophia Mihalyi[*,a], Irene Milani[a], Diego Romano[b], Silvia Donzella[b], Marion Sumetzberger-Hasinger[a], Felice Quartinello[*,a,c], Georg M. Guebitz[a]

[a]BOKU University, Vienna, Department of Agrobiotechnology, IFA-Tulln, Institute of Environmental Biotechnology, Konrad-Lorenz-Strasse 20, 3430 Tulln an der Donau, Austria

[b]Università degli Studi di Milano, Department of Food, Environmental, Nutritional Sciences (DeFENS), via Celoria 2, 20133 Milan, Italy

[c]acib GmbH, Konrad-Lorenz-Strasse 20, 3430 Tulln an der Donau, Austria

[*]Corresponding authors:
Felice Quartinello: felice.quartinello@boku.ac.at , +43 1 47654-97488
Sophia Mihalyi: sophia.mihalyi@boku.ac.at, +43 1 47654-97484

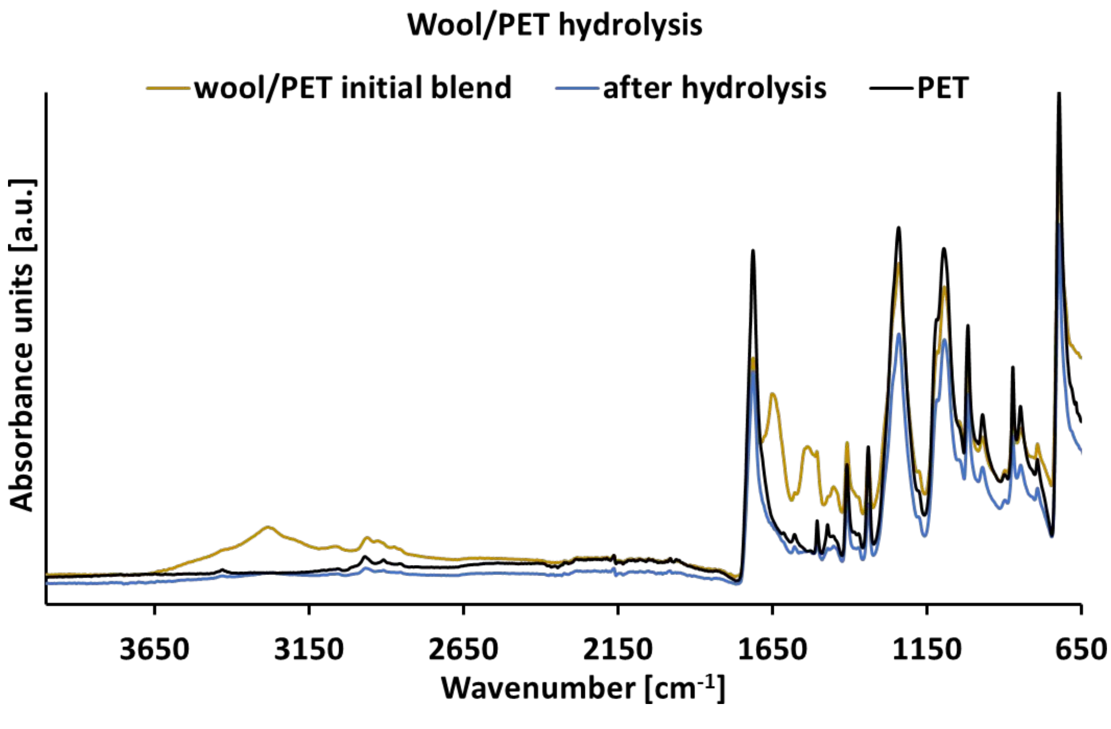

Figure S1 FTIR spectra after enzymatic hydrolysis of wool from WO/PET textile waste blends.

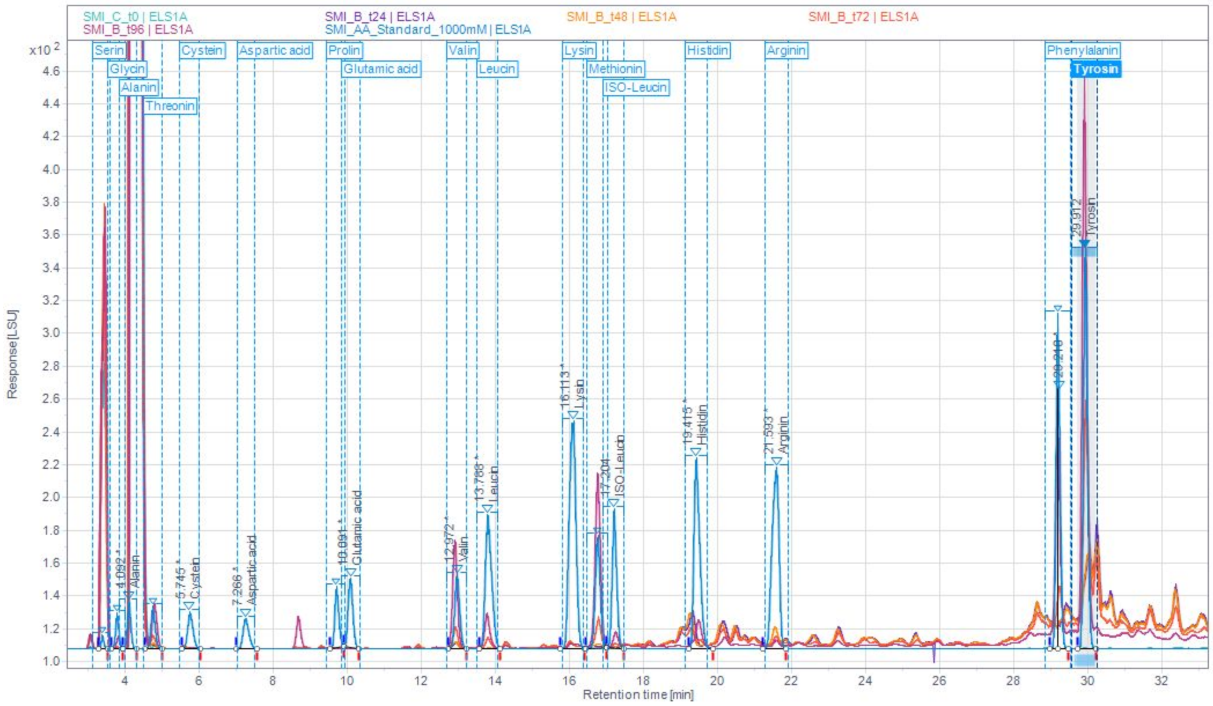

Figure S2 HPLC chromatogram from amino acid analysis of wool hydrolysis samples from timepoint 0 h (light blue), 24 h (dark purple), 48 h (orange), 72 h (red), 96 h (purple) and standards at 1000 µM concentration (blue) including 17 proteinogenic amino acids.

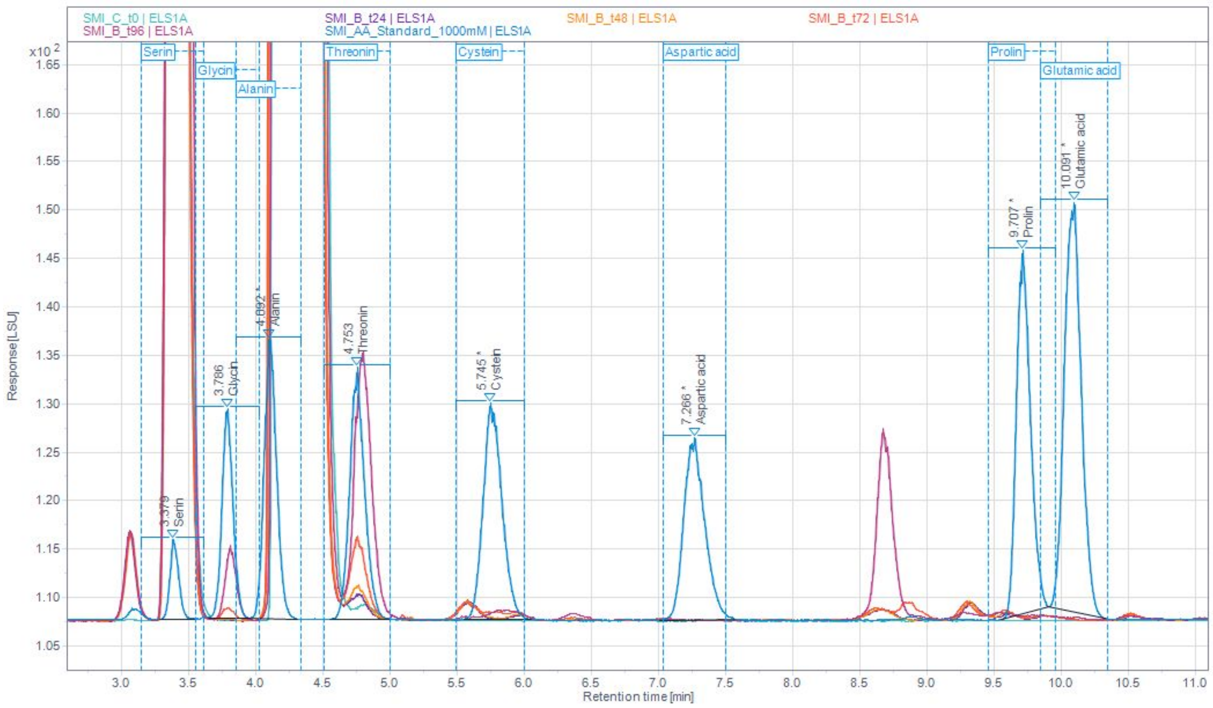

Figure S3 Zoom into HPLC chromatogram from amino acid analysis of wool hydrolysis samples from timepoint 0 h (light blue), 24 h (dark purple), 48 h (orange), 72 h (red), 96 h (purple) and standards at 1000 µM concentration (blue) including 17 proteinogenic amino acids.

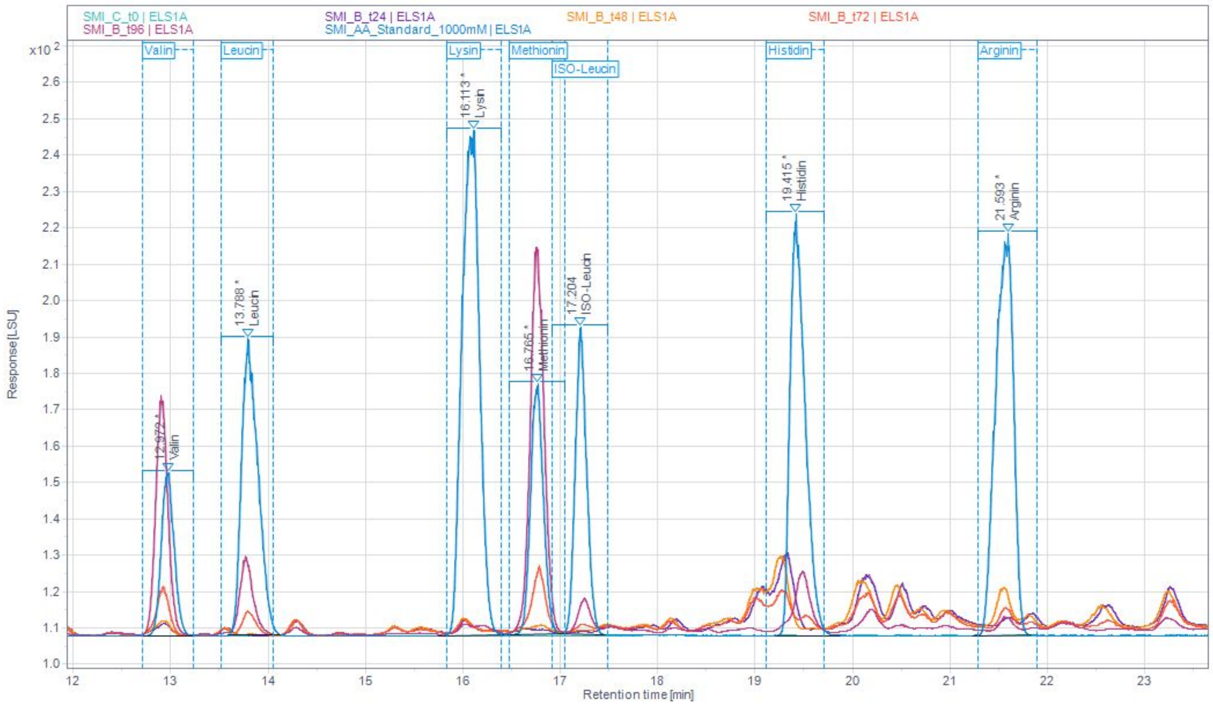

Figure S4 Zoom into HPLC chromatogram from amino acid analysis of wool hydrolysis samples from timepoint 0 h (light blue), 24 h (dark purple), 48 h (orange), 72 h (red), 96 h (purple) and standards at 1000 µM concentration (blue) including 17 proteinogenic amino acids.

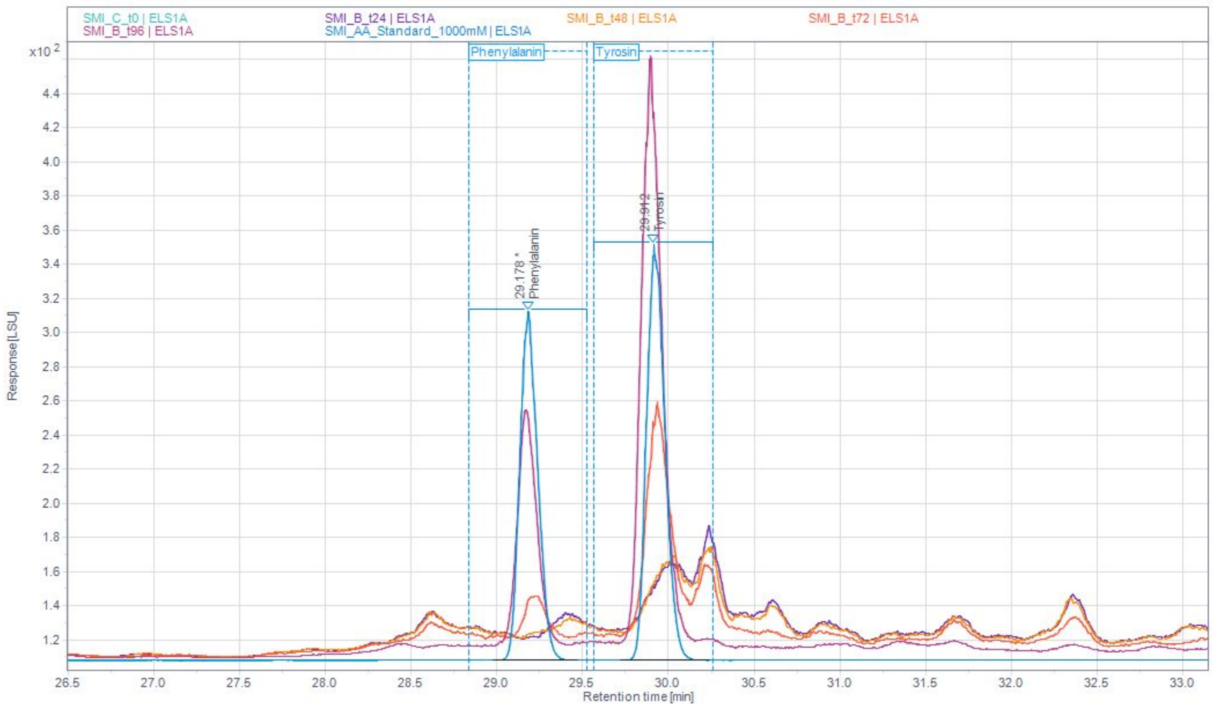

Figure S5 Zoom into HPLC chromatogram from amino acid analysis of wool hydrolysis samples from timepoint 0 h (light blue), 24 h (dark purple), 48 h (orange), 72 h (red), 96 h (purple) and standards at 1000 µM concentration (blue) including 17 proteinogenic amino acids.

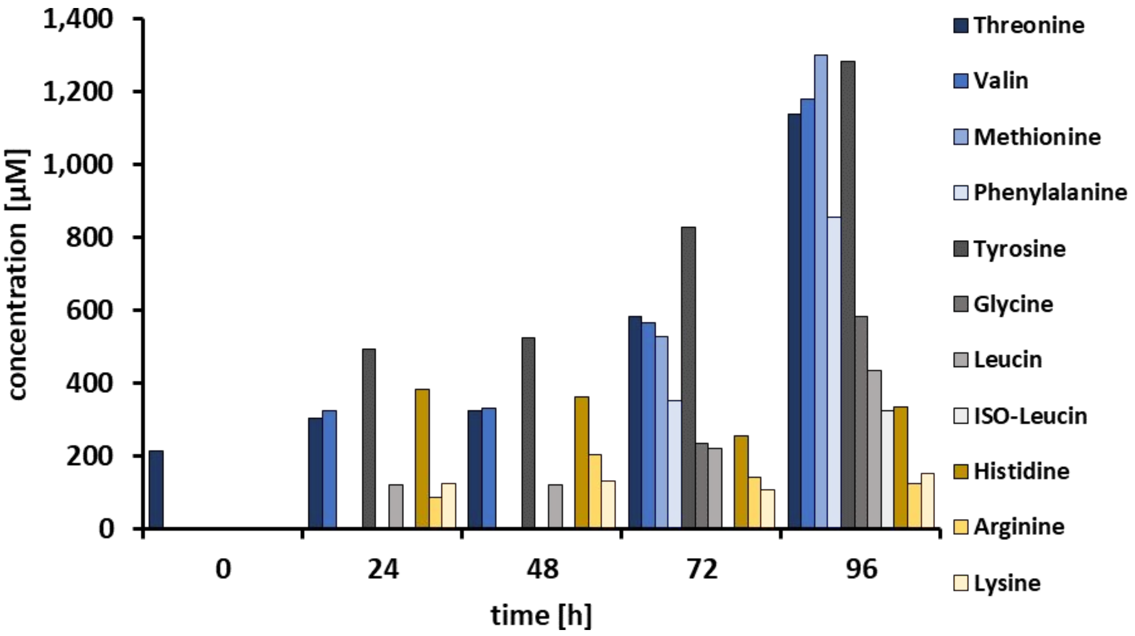

Figure S6 amino acid concentrations over time during wool hydrolysis from wool/PET blend.

Table S1 amino acid concentrations over time during wool hydrolysis from wool/PET blend in [µM] and [mg/L] and their sum.

Amino acid	0 h [µM]	24 h [µM]	48 h [µM]	72 h [µM]	96 h [µM]	0 h [mg/L]	24 h [mg/L]	48 h [mg/L]	72 h [mg/L]	96 h [mg/L]
**Threonine**	215	304	326	584	1138	26	36	39	70	136
**Valin**	0	325	333	566	1178	0	38	39	66	138
**Methionine**	0	0	0	527	1300	0	0	0	79	194
**Phenylalanine**	0	0	0	350	856	0	0	0	58	141
**Tyrosine**	0	494	523	827	1281	0	89	95	150	232
Glycine	0	0	0	234	584	0	0	0	18	44
Leucin	0	119	120	222	435	0	16	16	29	57
ISO-Leucin	0	0	0	0	323	0	0	0	0	42
Histidine	0	383	363	255	334	0	59	56	40	52
Arginine	0	86	205	141	125	0	15	36	25	22
Lysine	0	123	132	107	151	0	18	19	16	22
SUM	215	1834	2001	3813	7706	26	272	300	549	1080
